# Effects of Lysosomal Membrane Protein Depletion on the *Salmonella*-Containing Vacuole

**DOI:** 10.1371/journal.pone.0003538

**Published:** 2008-10-28

**Authors:** Everett A. Roark, Kasturi Haldar

**Affiliations:** 1 Department of Microbiology-Immunology, Northwestern University, Chicago, Illinois, United States of America; 2 Department of Pathology, Northwestern University, Chicago, Illinois, United States of America; Columbia University, United States of America

## Abstract

*Salmonella* is an intracellular bacterial pathogen that replicates within a membrane-bound vacuole in host cells. The major lysosomal membrane proteins 1 and 2 (LAMP-1 and LAMP-2) are recruited to the *Salmonella*-containing vacuole as well as *Salmonella*- associated filaments (Sifs) that emerge from the vacuole. LAMP-1 is a dominant membrane marker for the vacuole and Sifs. Its colocalization with both is dependent on a major secreted bacterial virulence protein, SifA. Here, we show that SifA is required for the recruitment of LAMP-2 and can be used as a second independent marker for both the bacterial vacuolar membrane and Sifs. Further, RNAi studies revealed that in LAMP-1 depleted cells, the bacteria remain membrane bound as measured by their association with LAMP-2 protein. In contrast, LAMP-2 depletion increased the amount of LAMP-1 free bacteria. Together, the data suggests that despite its abundance, LAMP-1 is not essential, but LAMP-2 may be partially important for the *Salmonella*-containing vacuolar membrane.

## Introduction

*Salmonella* infection causes millions of infections each year resulting in hundreds of thousands of deaths [1]. Serovar *S. typhimurium*, in particular, causes gastroenteritis in humans and enteric fever in animal hosts [2]. It is classified as a biodefense agent because it has the potential, and indeed, has been used to deliberately contaminate the food and water supply [3]. *Salmonella* species also now represents a leading cause of gram-negative bacterial meningitis in the developing world [1].

*Salmonella* is a facultative, intracellular, bacterial pathogen that replicates within a membrane-bound vacuole in both epithelial cells and in macrophages [4]. Infection initially occurs in the gastrointestinal tract as a result of ingesting contaminated foods. Once ingested, the bacteria cross the intestinal epithelial barrier by invading epithelial and M cells of the Peyer's patches [5]. Following the initial invasion, *Salmonella* serovars capable of causing systemic infection survive and replicates inside macrophages of the spleen and liver [5].

*Salmonella* is able to invade both phagocytic and non-phagocytic cells. Non-phagocytic cells are invaded by utilization of a Type III secretion system (TTSS) located on the *Salmonella* pathogenicity island -1 (SPI-1) [6]. Once the bacteria enter the cell, whether SPI-1 TTSS mediated or by phagocytosis, the *Salmonella* containing vacuole (SCV) becomes acidified and a second TTSS, located on *Salmonella* pathogenitiy island-2 (SPI-2), is activated [7]. The SPI-2 TTSS secreted effectors modify the host cell, preventing fusion of the SCV with lysosomes and allowing for bacterial replication within the vacuole [8].

A major SPI-2 effector, SifA, is known to be responsible for the stability of the SCV and formation of *Salmonella*-induced filaments (Sifs) [9]. Sifs are long, membranous extensions that radiate out from the SCV and are labeled by lysosome-associated membrane protein-1 (LAMP-1). Late in infection, Sifs are formed along microtubules once the bacteria start replicating [10]. In addition to Sif formation, SifA is required for the recruitment of LAMP-1 to the SCV [8], [11], and this is thought to be a major mechanism for membrane growth and stability at the SCV. Although late endosomal/lysosomal membrane proteins like the mannose 6-phosphate receptor are not found at the SCV [12], a second lysosomal membrane protein, LAMP-2, is also detected at the SCV [13]. However, it is not known whether analogous to LAMP-1, LAMP-2 recruitment also requires SifA or a distinct SPI-2 effector.

LAMP-1 and LAMP-2 are both type I transmembrane proteins. Each has a large lumenal domain which is highly glycosylated, and a short C-terminal cytoplasmic tail [14]. They share 37% amino acid sequence identity, but are distinct proteins which most likely diverged relatively early in evolution [15]. They both function in lysosome stability by protecting the lysosome membrane from hydrolytic damage [14], [15]. LAMP-2 also functions in chaperone-mediated autophagy [16] and immunity [17]. LAMP-1 is known to be more abundant than LAMP-2 [14], [15]. Although both proteins are well characterized in mammalian cells, their requirement and relative importance in establishing the SCV membrane remains unknown.

Here, we establish that although LAMP-1 is predominantly used as a membrane marker for the SCV, under normal conditions of intracellular infection *S. typhimurium* specifically and quantitatively associate with LAMP-2. Therefore, LAMP-2 can serve as a second marker of the SCV. By utilizing these two LAMPs as independent markers for the SCV and examining RNAi treated cells, we evaluated the relative contribution of each to the stability of the SCV membrane. These are the first studies investigating the requirement of resident lysosomal membrane proteins at the SCV.

## Results

### LAMP-2 is recruited to the *S. typhimurium* containing vacuole in infected cells

**Figure 1:**
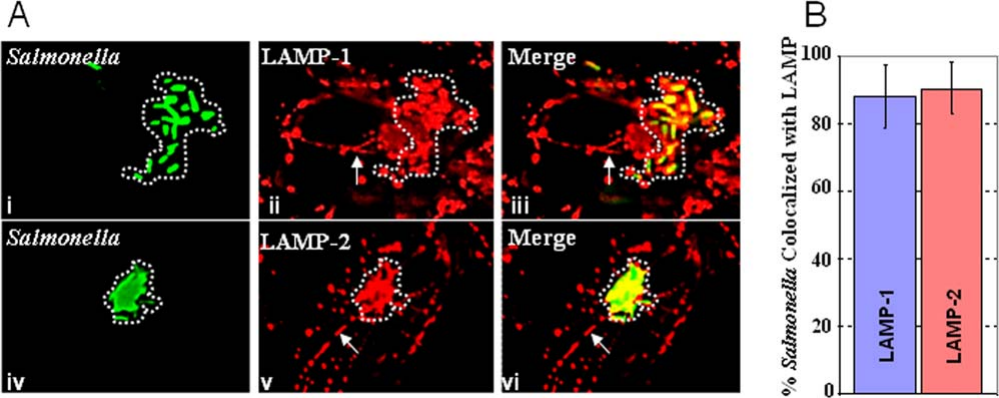
LAMP-2 localizes to the SCV and Sifs. A. HeLa cells were infected with wild type bacteria expressing GFP. 18 hours post-infection, the monolayers were fixed and immuno-stained to detect LAMP-1 (i–iii) or LAMP-2 (iv–vi). Green bacteria co-localized with LAMP-1 and LAMP-2, suggesting that both are recruited to the SCV (indicated by the dotted line). Sifs, which are marked by arrows, are also labeled by LAMP-1 and LAMP-2. B. The extent of LAMP-2 labeling of the SCV is comparable to that seen for LAMP-1. Volocity image analysis software was used to quantitate the colocalization of GFP-*Salmonella* and LAMP-1 or LAMP-2. A two-tailed P value was obtained from the Volocity quantitation using the Student's T test. The P value is = 0.56 which indicates there is no significant difference between the colocalization of *Salmonella* with LAMP-1 or LAMP-2.

Multiple studies have shown that LAMP-1 is recruited by the SCV and that LAMP-1 is localized to Sifs [2], [8], [9]. However, the relationship between LAMP-2 and the intravacuolar bacteria has been less well investigated. Several papers have indicated that LAMP-2 is localized to the SCV, but a comparative, side-by-side analysis of LAMP-2 and LAMP-1 recruitment to the SCV and Sifs has not been undertaken. We, therefore, infected HeLa cells with *Salmonella* (expressing GFP to ease detection of bacteria) and allowed the infection to proceed for 18 hours. After this period, the cells were fixed and immuno-stained to detect LAMP-1 or LAMP-2, and subsequently imaged. As expected, LAMP-1 was detected surrounding intracellular bacteria (at the SCV, Fig. 1A) and on Sif membranes (indicated by arrow in Fig. 1A, panels ii and iii). LAMP-2 was also associated with intracellular bacteria and with Sifs (Fig. 1A, iv–vi). On average, 80–100% of intracellular bacteria were labeled by both LAMP-1 and LAMP-2 (Fig. 1B) suggesting that both proteins are prominently associated with the SCV and Sifs. This suggested that like LAMP-1, LAMP-2 may also be used as a major marker of SCVs and Sifs.

### LAMP-2 recruitment to the SCV is reduced in the absence of SifA

As indicated earlier, previous studies have demonstrated that the *Salmonella* SPI-2 effector protein, SifA, is required for LAMP-1 recruitment to the SCV [18]. Since LAMP-1 and LAMP-2 are related proteins, we were interested in determining whether SifA is required for LAMP-2 recruitment to the SCV. To do this, we wanted to examine Δ*sifA* mutants, but prior work has shown that these mutants escape from the vacuole after the SPI-2 TTSS is activated [19]. However, it has also been shown that if *sifA* is knocked-out in conjunction with its antagonist, the SPI-2 effector *sseJ*, the bacteria remain in the SCV [20]. In light of this, LAMP-2 recruitment was compared between the double mutant, *ΔsseJ/ΔsifA*, relative to the *ΔsseJ* mutant.

**Figure 2:**
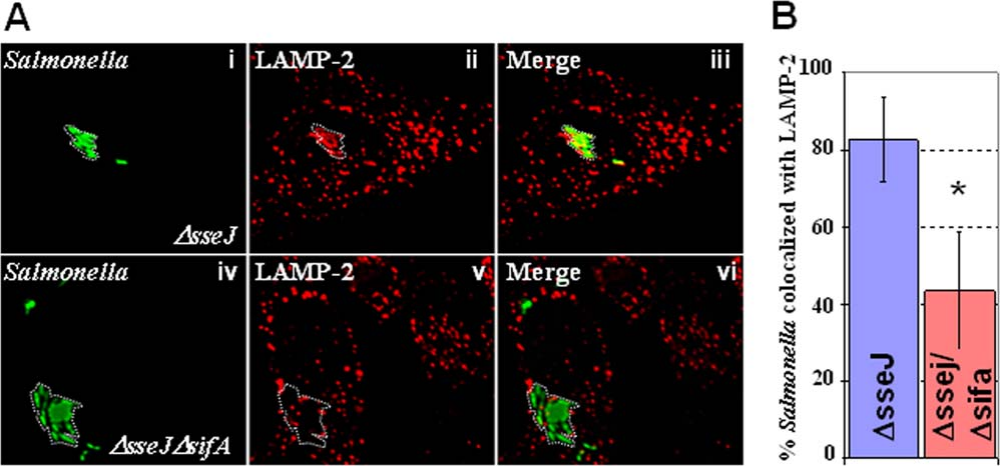
LAMP-2 recruitment is mediated by SifA. A. HeLa cells were infected with *ΔsseJ* (i–iii) or Δ*sseJ*/Δ*sifA* (iv–vi) mutants (green). 18 hours post-infection, the monolayers were fixed and imunno-stained for LAMP-2 (red). Δ*sseJ* bacteria colocalize with LAMP-2 (i–iii). *ΔsseJ/ΔsifA* mutants (green; iv–vi), show significantly reduced co-localization with LAMP-2. B. Volocity image analysis software was used to quantitate association of bacteria with LAMP-2 in *ΔsseJ/ΔsifA* and *ΔsseJ* mutants. A two-tailed P value was obtained from the Volocity quantitation using the Student's T test. The P value is 0.0001 which indicates there is significant difference between the colocalization of LAMP-2 with *ΔsseJ* versus *ΔsseJ/ΔsifA*.

To determine if SifA was required for the recruitment of LAMP-2, HeLa cells were infected with *ΔsseJ/ΔsifA* or *ΔsseJ* bacteria, fixed and immuno-stained to detect LAMP-2, and subsequently imaged (Materials and Methods). As shown, *ΔsseJ* mutants recruited a high level of LAMP-2 to the SCV (Fig. 2A i–iii), which was comparable to that seen with wild type bacteria (Fig. 1). In contrast, the *ΔsseJ/ΔsifA*-*Salmonella* showed significantly lower levels of LAMP-2 at the SCV (Fig. 2A, iv–vi). Quantitative analyses revealed a significant decrease (P = 0.0001) of 40% in the amount of LAMP-2 recruited to the SCV of *ΔsseJ/ΔsifA* mutants relative to *ΔsseJ* (Fig. 2b). These results suggest that SifA is required for a significant fraction of LAMP-2 recruitment to intracellular bacteria. SifA is also required for the recruitment of LAMP-1.

### LAMP-2 knockdown promotes reduction of LAMP-1 at the SCV membrane

**Figure 3:**
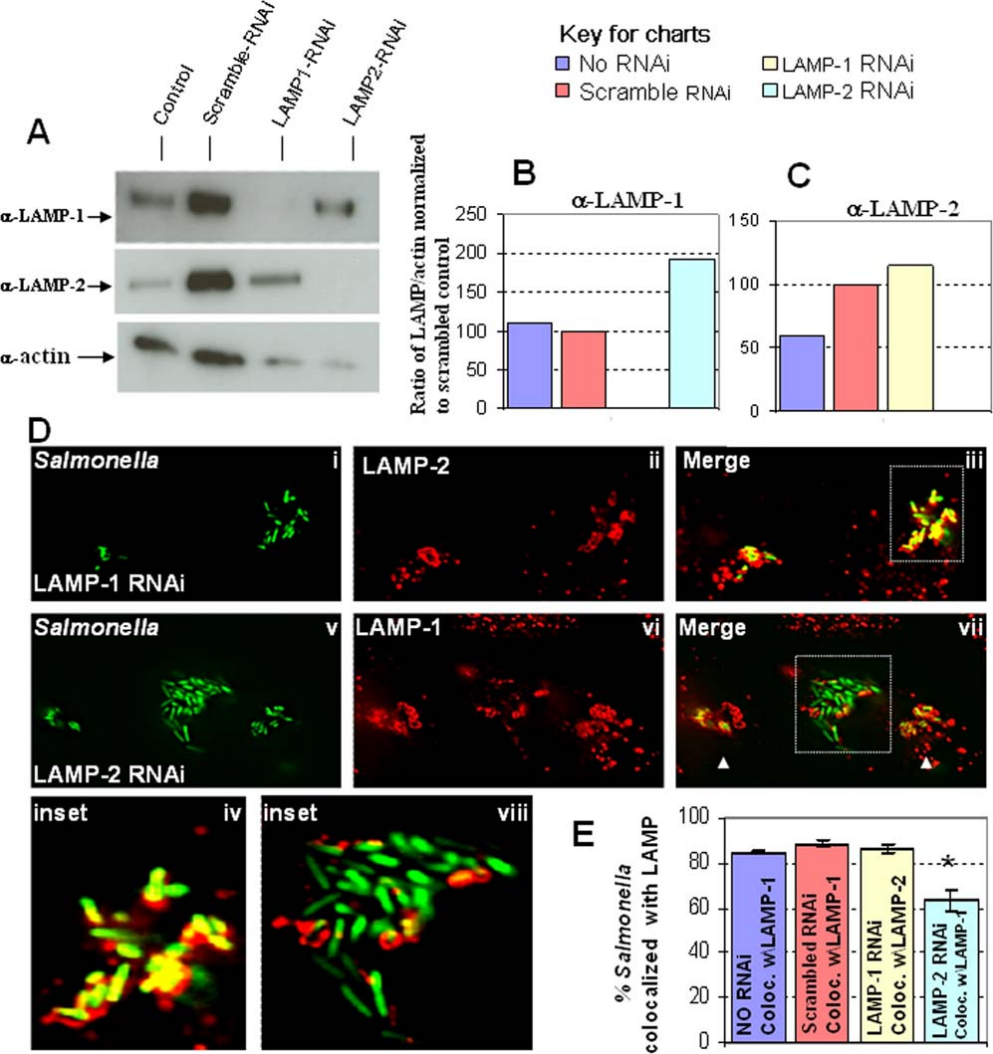
LAMP-2 knockdown increases prevalence of LAMP-1 depleted bacteria. A. RNAi treated HeLa cells were lysed 48 hours after RNAi treatment was initiated. Equal volumes of whole cell lysates were analyzed by Western for LAMP-1, LAMP-2 and actin (used as a loading control). B. LAMP-1:actin ratio relative to that in scrambled RNAi samples (set to 100) C. LAMP-2:actin ratio relative to that in scrambled RNAi samples (set to 100). The data in A–C show that LAMP-1 RNAi specifically knocks down LAMP-1 but not LAMP-2. In addition, LAMP-2 RNAi specifically knocks down LAMP-2 and not LAMP-1 D. HeLa cells were transfected with RNAi oligos to knockdown either LAMP-1 (i–iv) or LAMP-2 (v–ix). 48 hours after RNAi treatment, the cells were infected with *Salmonella* for 6 hours. The cells were then fixed and immuno-stained. LAMP-1 knockdown cells were immuno-stained for LAMP-2 and revealed consistent colocalization of *Salmonella* with LAMP-2 (iv). However, immuno-staining of LAMP-2 knockdown cells for LAMP-1 revealed reduced association of LAMP-1 in a subset of bacterial clusters (viii). Clusters of bacteria containing normal levels of LAMP-2 are shown by arrow heads. E. Volocity quantitation of bacteria association with LAMP-containing membrane in knockdown cells. A two-tailed P value was obtained from the Volocity quantitation using the Student's T test. Asterisk indicates the P value is less than 0.001 which indicates that the difference is significant.

In order to determine the relative importance of LAMP-1 and LAMP-2 to *Salmonella* infection, we used RNAi to knockdown levels of these proteins in cells. Cytoplasmic actin was used as a measure of total cellular protein and cell growth. As shown in Fig. 3A, scrambled RNAi oligos did not significantly alter total levels of actin, suggesting that they did not affect cell growth. Unexpectedly these scrambled oligos increased LAMP-1 and LAMP-2 levels in cells. In contrast, specific oligonucleotides designed to knock down LAMP-1 and LAMP-2 consistently showed reduced actin levels. These cells were nonetheless adherent and viable, suggesting they were slowed in growth. However, despite reduced growth cells knocked down in LAMP-1 displayed LAMP-2 levels that were modestly increased relative to control incubations. Similarly cells knocked down in LAMP-2 displayed LAMP-1 levels that were close to those seen in control incubations. Importantly actin levels in cells knocked down in LAMP-1 or LAMP-2 were comparable and this enabled us to ascertain the effect of knockdown of each protein on the recruitment of the other, to the SCV. To facilitate the analysis we determined levels of LAMP-1 and LAMP-2 detected normalized for actin (Fig. 3B–C) for all samples. In summary the data in Fig. 3A–C suggested that, knockdown of LAMP-2 did not abrogate LAMP-1 levels in cells and conversely knock down of LAMP-1 did not abrogate LAMP-2 levels in cells. Since we showed in Fig. 1 that both LAMP-1 and LAMP-2 could be used to mark the SCV, the data in Fig. 3A–C suggested that we could investigate the consequence of depleting each LAMP to the stability of the SCV by using the other to mark the SCV.

**Figure 4:**
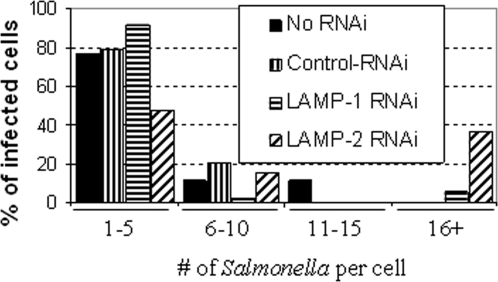
LAMP-1 depleted *Salmonella* in LAMP-2 knockdown cells proliferate at an accelerated rate. Bar plot shows the number of *Salmonella* per infected cell. HeLa cells were transfected with RNAi oligos to knockdown LAMP-1 or LAMP-2. 48 hours after RNAi treatment, the cells were infected with GFP-*Salmonella* for 6 hours, then fixed and immuno-stained. The amount of bacteria per infected cell was counted and the results shown here. The cells that contained more than 20 bacteria were the ones in which the *Salmonella* were not contained in LAMP-1 vacuoles.

Thus, cells knocked down in LAMP-1 or LAMP-2, along with controls, were infected with *S. typhimurium*, then fixed and immuno-stained (Fig. 3D). In untreated cells or cells treated with control RNAi oligonucleotides, bacteria were quantitiatively associated with both LAMP-1 and LAMP-2 (Fig. 3D panels i–iii and Fig. 3E). Furthermore when LAMP-1 was knocked down, bacteria continued to quantitatively associate with LAMP-2. However, when LAMP-2 was knocked down, approximately 40% of bacteria were no longer positive for LAMP-1 (Fig. 3D, panels vii–inset, viii and Fig. 3E). These LAMP-1-negative bacteria were detected in large clusters containing 20+ bacteria.(Fig. 4). The other 60% remained associated with LAMP-1 (Fig. 3D, arrowheads, panel vi–vii, Fig. 3E) in clusters of five or less bacteria (Fig. 4). These data suggest that bacteria in large clusters reside in a different vacuolar compartment or may be free in the cytoplasm. Regardless, they proliferate at an accelerated rate suggesting that their intracellular environment is functionally altered. Together, the data in Figures 3 and 4 suggest that although LAMP-1 is a major membrane marker for the SCV, its loss in cells does not affect the stability of the SCV. In contrast, LAMP-2 depletion measurably reduced detection of the SCV marker LAMP-1, suggesting LAMP-2 contributes to either the composition and function, or the stability of the SCV membrane.

## Discussion

The SCV is a lysosmal-like compartment in that it recruits major lysosomal membrane proteins, LAMP-1 and LAMP-2 [8], [11]–[13]. However, it excludes digestive, soluble enzymes, such as cathepsins, which are usually found in the lumen of the lysosome. The SCV also excludes membrane-bound receptors, such as the mannose-6-phosphate receptor that deliver cathepsins and other soluble cargo to the lysosomes [12]. Characterization of the recruitment of LAMP-2 along with that of LAMP-1 enabled each to be used as a marker for the SCV, as well as testing (in RNAi experiments) whether each was important to the SCV membrane.

Numerous effectors secreted by *S. typhimurium* modify the host cell and the functions of each effector appear to be distinct. Examples include SseG, which is thought to be important for recruitment of the Golgi; SseJ, which esterifies host cholesterol; SipB, which binds and activates caspase-1, SopE1 and SopE2 act as G-nucleotide exchange factors; and SipA and SipC, which directly bind actin and modulating bacterial uptake [21]–[25].

SifA influences the position of the SCV, is necessary for Sif production, and is required for the recruitment of LAMP-1 to these membranes [9], [26]. Our data suggest that SifA is also required for the recruitment of LAMP-2, possibly explaining why SifA is essential for membrane stability of the SCV. In addition, our data implicate the presence of a cellular mechanism engaged by SifA that enables the recruitment of LAMP-1 and LAMP-2, while circumventing mannose-6-phosphate receptor trafficking between the Golgi and lysosomes. Further, although LAMP-1 is the major lysosomal membrane protein in cells and the dominant protein marker of the SCV, we find that it is not essential for the SCV membrane. In contrast, LAMP-2 which is present at three fold lower levels, does contribute in a measurable way to the composition and/or maintenance of a stable SCV.

In mouse models, LAMP-1/LAMP-2 double knockout is embryonically lethal [27]. However, the single knockouts of LAMP-1 or LAMP-2 are both viable [27]–[29]. The LAMP-1 knockout mice display a mild phenotype, and apart from mild regional astrogliosis and altered immunoreactivity against cathepsin D in the brain, all tissues in the mouse are similar to controls [28]. In addition, their lysosomes are similar to control mice in both form and function [28]. In contrast, the LAMP-2 knockout mice display a more severe phenotype. Fifty percent of the mice die by 6 weeks [29]. In these mice, the knockout of LAMP-2 leads to cardiomyopathy and an increase in the amount of autophagy/lysosome compartments in many tissues [30]. However, these compartments are less efficient at protein degradation than their wild-type counterparts [30]. These symptoms mirror those of human Danon disease which is attributed to mutations in LAMP-2 [30], [31]. Thus, despite similarities in location, LAMP-2 function in the host is likely to be distinct from that of LAMP-1. Our data support that LAMP-2 provides a more critical function in formation, composition and/or maintainence of the SCV.

## Materials and Methods

### Antibodies and reagents

Anti-LAMP-1 mouse monoclonal antibody, H4A3, was purchased from Iowa Developmental Studies Hybridoma Bank and used at 1∶1000. Anti-LAMP-2 mouse monoclonal antibody, H4B4, was purchased from Iowa Developmental Studies Hybridoma Bank and used at 1∶1000. Anti-actin mouse monoclonal antibody, MAB1501, was purchased from Millipore Corporation and used at 1∶2500. Rhodamine-conjugated goat IgG fraction raised against mouse IgG was purchased from ICN/Cappel (Solon, OH).

### Cell culture

HeLa cell lines (ATCC) were grown in Modified Eagles Medium (MEM) (ATCC) supplemented with 10% (v/v) fetal calf serum. The cell lines were maintained at 37°C in 5% CO_2_, passaged every 2–3 days, and used for experiments at a passage number less than 20.

### Bacterial strains and growth conditions

The *S. typhimurium* wild type strain SL1344 (ATCC) was used for all experiments and construction of mutants, except where otherwise noted. Expression of GFP in this strain and additional strains was accomplished by the introduction plasmid pFPV25.1 [32]. The Δ*ssaT* mutant has been described previously [33], [34], and was a gift from Dr. Bradley Jones (University of Iowa). Knockouts in *sseJ* and *sifA* were constructed using the method developed by Datsenko *et al.* [35], a PCR-based knockout strategy based on the Red system [36]. For infections, bacteria were grown to late log phase in LB containing 0.3 M NaCl without shaking. Optical density measurements at 600 nm were taken to calculate bacterial cell density. An optical density of 1.0 was approximately equal to 1.3×10^9^ CFU/ml. Ampicillin (50 µg/ml), kanamycin (50 µg/ml), and streptomycin (50 µg/ml) were used for selection of bacterial strains.

### Cell infections

HeLa cells were seeded at 3×10^5^ per well in a 24-well tissue culture plate, and were allowed to grow for at least 12 hours prior to infection. Bacterial cultures were washed and diluted into serum free MEM, added to Hela cells at a multiplicity of infection of 100, and subjected to centrifugation at 1000 rpm for 5 minutes. Following centrifugation, the cells were placed at 37°C for 1 hour, washed three times with serum free medium, and then incubated in 100 µg/ml of gentamicin in 10% FBS-MEM for 60–90 minutes. After this time, the concentration of gentamicin was adjusted to 10 µg/ml for the remainder of the experiment.

### Indirect immunofluorescence assays

HeLa cells were grown on glass coverslips, infected with bacteria, fixed, and then processed for immunofluorescence [37]. Primary antibodies to the following markers from indicated sources were used at the indicated dilutions LAMP-1 (H4A3, mouse, Iowa Developmental Studies Hybridoma Bank 1∶1000) and LAMP-2 (H4B4, mouse, Iowa Developmental Studies Hybridoma Bank 1∶1000). The secondary antibody, rhodamine conjugated goat anti mouse IgG, was used at dilutions of 1∶800 (ICN/Cappel, Solon, OH). Samples were examined by Deltavision deconvolution fluorescence microscopy using standard UV, FITC and rhodamine, Cy-5, GFP, RFP filter sets.

### Deconvolution fluorescence microscopy and quantitative image analyses

Fluorescence microscopy and digital image collection were performed using an Olympus IX inverted fluorescence microscope and a Photometrix cooled CCD camera (CH350/LCCD) driven by DeltaVision software from Applied Precision Inc. (Seattle, WA). Images used for Fluorescence quantification were obtained with a 60×, NA 1.4 objective. Briefly, 300-nm optical sections were taken through the depth of the cell, and DeltaVision software (softWoRx) was used to deconvolve these images. For colocalization quantitation, a stack of TIFFS for each image was generated and exported to Volocity software (Improvision, Coventry, UK). For monolayers infected with wild-type or mutant bacteria, values were obtained for the total cholesterol in 20 cells. The total amount of colocalization in each cell was quantitated by measuring and integrating the intensity values over all selected voxels in the Filipin UV channel. To obtain values for the SCV-associated marker in these cells, the bacterial cluster in the best focal plane was selected using the freehand selection tool and the software was then allowed to extend the selection through all z-sections in both channels.

### Knockdown experiments using siRNA

Small interference RNA (siRNA) against LAMP-1 (5′-AGAAAUGCAACACGUUA-3′) and LAMP-2 (5′-GCUGUGCGGUCUUAUGCAU-3′) along with RNAi negative control (5′-AAACTTGTCGACGAGAAGCAA-3′) were purchased from Invitrogen (Carlsbad, CA). HeLa cells were transfected with the siRNA targeting LAMP-1, LAMP-2 or nonspecific control RNA (100 pmol/ml) using Oligofectamine according to the manufacturer's instructions. Knockdown of LAMP-1 and LAMP-2 was confirmed by Western blot analysis.
